# Lipschütz ulcers after AstraZeneca COVID-19 vaccination^☆^

**DOI:** 10.1016/j.abd.2022.09.010

**Published:** 2023-04-25

**Authors:** Maria Bracho-Borro, Graciela Guzmán-Perera, Mario Magaña

**Affiliations:** a Dermatology Department, General Hospital of Mexico Dr. Eduardo Liceaga, Mexico City, Mexico; b Dermatology Department, Hospital Ángeles del Pedregal, Mexico City, Mexico; c Dermatology Department, General Hospital of Mexico Dr. Eduardo Liceaga, Mexico City, Mexico

Dear Editor,

Acute vulvar aphthous ulceration or Lipschütz ulcer is a rare non-sexually acquired condition which is characterized by the sudden onset of painful genital ulcers. It doesn’t have a clear etiology; therefore, its diagnosis is challenging. The usual course is self-resolution without relapses and scarring. There have been six cases reported following COVID-19 vaccination.^1^, ^2^

## Case Report

**Figure 1:**
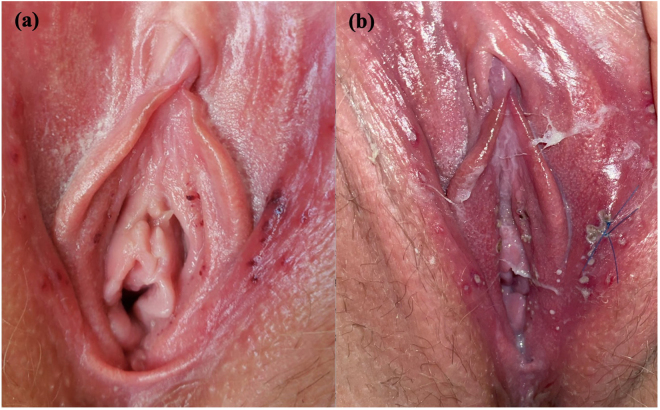
Progression of vulvar aphthosis. (A) Initial clinical presentation, with millimetric, shallow, purple-red, ulcerations, (B) Day 1 follow-up. Labia majora and minora with edema and erythema. Ulcerations with purulent center

**Figure 2:**
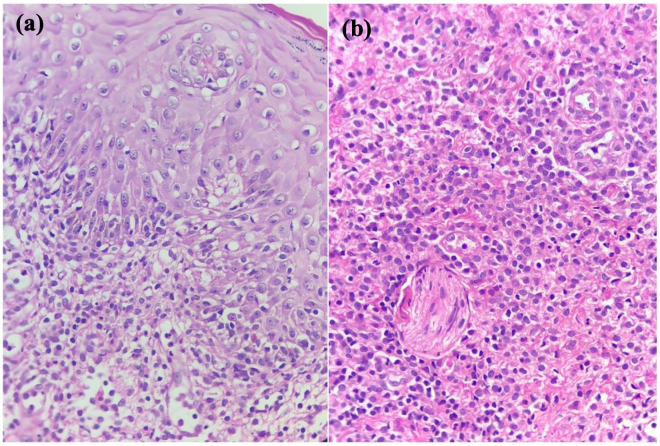
Biopsy of one ulcer stained with Hematoxylin & eosin, 40x. (A) Vulvar mucosa ulceration and spongiosis. (B) Dense mixed inflammatory infiltrate of lymphocytes, histiocytes, and scarce monocytes in all the stroma.

A 27-year-old healthy, sexually active woman received her third dose of the COVID-19 vaccine, the first two were Pfizer-BioNTech and this one was AstraZeneca (Vaxzevria) vaccine. She complained of acute onset of burning pain in labia majora and swelling 24 hours after receiving the vaccine, accompanied by fever and body aches. Physical examination revealed multiple millimetric, shallow, purple-red, painful ulcerations in labia majora, labia minora, and vaginal introitus (Fig. 1). The patient had no previous history of genital ulcers. Oral mucosa and oropharynx were clear. At 24 hours follow up she reported worsening symptoms complaining of difficulty sitting down. Upon examination, the patient had more ulcerations with yellow and gray covering with surrounding erythema and edema, some with the purulent center. A biopsy of one ulcer was taken and tissue PCR for Herpes Simplex Virus (HSV) 1/2, Varicella-Zoster Virus (VZV), and cytomegalovirus was negative, as well as serologic IgG and IgM for HSV 1/2 and VZV. HIV serology was not reactive, genital swab culture, VDRL, COVID antigen test, and antinuclear antibodies were all negative. The biopsy showed epithelium ulceration, spongiosis, and dense inflammatory infiltrate of lymphocytes and histiocytes, there was no trace of vasculitis or viral infection (Fig. 2). The patient was diagnosed with vulvar aphthous ulcers. She was treated with hydrocortisone 1% and pain control. Ulcers resolved within 9 days. At the 6 month follow-up, she had not presented a recurrence of the lesions.

## Discussion

Although the exact etiology of Lipschütz ulcer remains unclear, it likely involves an immunologic response to an infection or other source of inflammation. Most cases are idiopathic with a negative infectious workup.^1^ There are six case reports of vulvar aphthous ulcers following COVID-19 vaccination, four after Pfizer-BioNTech and one after AstraZeneca.^1^, ^2^ This is the second one reported in the literature after the AstraZeneca vaccination. The diagnosis was challenging since the patient was sexually active. Sexually transmitted diseases were tested for and excluded. After discarding infectious diseases, the main differential diagnosis was Behçet syndrome, however, the biopsy did not show signs of vasculitis.^3^ Lipschütz’s ulcers were firstly attributed to virgin women, and later to patients with an absence of sexual contact in the previous three months. However, in a study conducted in Brazil, 86 out of 98 patients were diagnosed with Lipschütz ulcers when expanding criteria to any age and sexual activity.^4^ Another retrospective analysis of 33 cases found that 84.8% of the patients had had their sexual debut.^5^

Several cutaneous reactions to the COVID-19 vaccination have been reported. This case demonstrates a potential association between this vaccine and the development of a vulvar aphthous ulcer. Clinicians, especially dermatologists, gynecologists and pediatricians should be aware of the possible risk of this disease after COVID-19 vaccination.

## Financial support

None declared.

## Authors’ contributions

Maria Bracho-Borro: Writing of the manuscript or critical review of important intellectual content; effective participation in the research guidance; intellectual participation in the propaedeutic and/or therapeutic conduct of the studied cases; critical review of the literature.

Graciela Guzmán-Perera: Writing of the manuscript or critical review of important intellectual content; effective participation in the research guidance, intellectual participation in the propaedeutic and/or therapeutic conduct of the studied cases.

Mario Magaña: Data collection, analysis, and interpretation; intellectual participation in the propaedeutic and/or therapeutic conduct of the studied cases.

## Conflicts of interest

None declared.
